# The Role of mtDNA Mutations in Atherosclerosis: The Influence of Mitochondrial Dysfunction on Macrophage Polarization

**DOI:** 10.3390/ijms26031019

**Published:** 2025-01-25

**Authors:** Evgeniya I. Fedotova, Alexey V. Berezhnov, Daniil Y. Popov, Elena Y. Shitikova, Andrey Y. Vinokurov

**Affiliations:** 1 Institute of Cell Biophysics of the Russian Academy of Sciences, Pushchino 142290, Russia; delf-fenka@rambler.ru (E.I.F.); alexbereg56@gmail.com (A.V.B.); 2 Cell Physiology and Pathology Laboratory, Orel State University, Orel 302026, Russia; rennda@yandex.ru (D.Y.P.); sti.bium@yandex.ru (E.Y.S.)

**Keywords:** mtDNA, mutations, macrophages, polarization, inflammation, atherosclerosis

## Abstract

Atherosclerosis is a complex inflammatory process associated with high-mortality cardiovascular diseases. Today, there is a growing body of evidence linking atherosclerosis to mutations of mitochondrial DNA (mtDNA). But the mechanism of this link is insufficiently studied. Atherosclerosis progression involves different cell types and macrophages are one of the most important. Due to their high plasticity, macrophages can demonstrate pro-inflammatory and pro-atherogenic (macrophage type M1) or anti-inflammatory and anti-atherogenic (macrophage type M2) effects. These two cell types, formed as a result of external stimuli, differ significantly in their metabolic profile, which suggests the central role of mitochondria in the implementation of the macrophage polarization route. According to this, we assume that mtDNA mutations causing mitochondrial disturbances can play the role of an internal trigger, leading to the formation of macrophage M1 or M2. This review provides a comparative analysis of the characteristics of mitochondrial function in different types of macrophages and their possible associations with mtDNA mutations linked with inflammation-based pathologies including atherosclerosis.

## 1. Introduction

**Figure 1:**
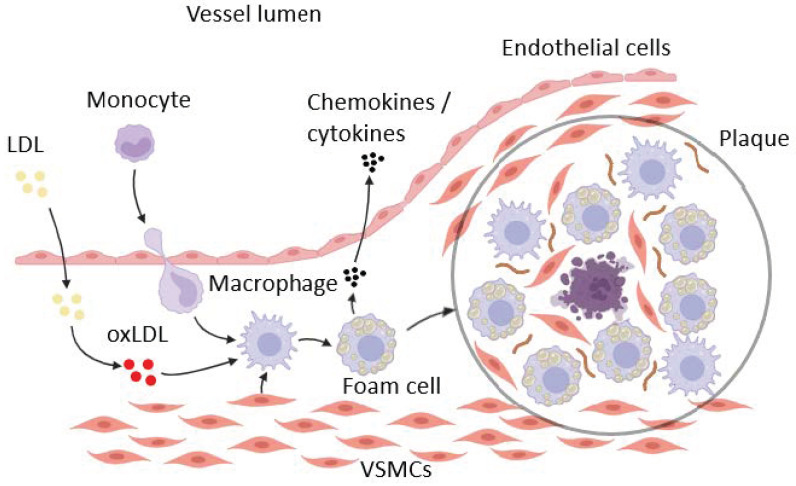
The role of macrophages in atherosclerosis plaque progression.

Atherosclerosis is a pathological process involved in many cardiovascular diseases including ischemic heart disease, cerebrovascular disease, atherosclerosis of the aorta, peripheral vascular disease, myocardial infarction, heart failure, and stroke, which are summarily the most common causes of global mortality [1,2,3,4]. Firstly described as a primarily metabolic disorder, now atherosclerosis is considered a widely studied complex inflammatory process (Figure 1) [2,5,6,7,8,9,10,11,12,13,14,15,16,17,18,19,20]. Despite the well-known leading role of low-density lipoproteins (LDLs) [21], triggers of atherosclerosis are being discussed. Impairment of the vascular wall of the arteries seems to be one of the most early stages of the pathology [11,22,23]. Subtle and focal physical or chemical injury is mostly associated with age-related processes, which result in weakened contacts between endothelial cells and facilitate the transport of LDL particles through the endothelial wall into the intima with subsequent forming of “lipid bands” [11,24]. Activated endothelial cells play the role of the primary source of cytokines, chemokines, and adhesion molecules, such as MCP-1, ICAM-1, VCAM-1, E-selectin, and P-selectin, facilitating the migration of leukocytes into the vessel intima [15,25]. Increased activity of myeloperoxidase, lipoxygenase, cyclooxygenase, cytochrome P450, NADPH oxidases (NOXs), xanthine oxidase, and the reactive oxygen species (ROS)-producing mitochondrial electron transport chain (ETC) with decreasing antioxidant capacity lead to LDL oxidation [14,18]. Engulfment of oxidized LDL particles (oxLDLs) by monocytes and polarized macrophages as well as by activated and transdifferentiated vascular smooth muscle cells (VSMCs) and, to a lesser extent, by other cell types leads to the formation of foam cells becoming a secondary source of massive cytokine release and a main cellular component of newly formed atherosclerotic plaque [22,26,27]. Size and stability of the plaque are the critical parameters of developing pathology. A large lipid core with a high level of necrosis and incomplete apoptosis or other programmed cell death pathways as well as a thin fibrous sheath, ongoing inflammation, and increased degradation of the extracellular matrix by metalloproteinases are the signs of vulnerable plaques, which lead to vessel thrombosis after rupture [14,16,22,28]. In about 30% of cases, vulnerable plaques can undergo erosion-like degradation [8,29,30]. Stable-growing plaques containing insoluble fibers based on type I collagen significantly impair blood circulation, leading to arterial narrowing and tissue ischemia [22,31,32].

Progression and resolution of inflammatory diseases including atherosclerosis are largely determined by immune cell activity, with macrophages playing the leading role [33,34,35,36,37,38,39,40]. On the one hand, pro-inflammatory cytokine-producing activity and transformation into foam cells create conditions of plaque growth; on the other hand, anti-inflammatory cytokine release and efficient efferocytosis lead to the removal of dead cells with stabilization and decrease in plaque [41,42,43]. Another aspect of macrophage influence is linked with atherosclerotic calcification [39].

**Figure 2:**
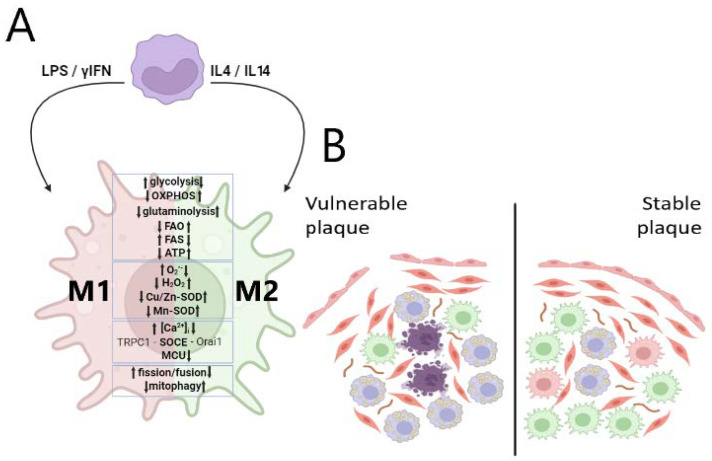
Macrophage plasticity and its role in atherosclerosis plaque stability. (**A**) External stimuli-based macrophage polarization with the formation of M1 and M2 cell types with differences in the phenotypic profile, including basal metabolic processes, redox balance, calcium homeostasis maintenance, and mitochondrial dynamics. (**B**) The difference in structure and stability of atherosclerosis plaques with the predominance of M1 or M2 macrophages (LPS—lipopolysaccharide; FAO—fatty acid oxidation; FAS—fatty acid synthesis; MCU—mitochondrila calcium uniporter; OXPHOS—oxidative phosphorylation; TRPC1—transient receptor potential canonical channel 1; SOCE—store-operated calcium entry; SOD—superoxide dismutase; and Orai1—calcium release-activated calcium channel protein 1).

Most of the arterial wall macrophages are polarized from blood-circulating monocytes recruited to adhesion molecules (such as VCAM-1), which are expressed by endothelial cells [7,14]. The smaller part of macrophages with a relatively high proliferation activity is derived from embryonic tissues [36,44]. It has been reported that a resident macrophage type from the circulating monocytes migrates into the aorta in the first year after birth [44]. Well-known macrophage metabolism plasticity leads to the formation of a heterogeneous cell population, which is divided into two groups: pro-inflammatory and anti-inflammatory cells [6,22,45]. Subsequent studies in vivo showed the oversimplification of such differentiation and made it possible to further subdivide macrophages of both groups (pro-inflammatory—M1, M4, and Mox and anti-inflammatory—M2a, M2b, M2c, M2d, M(hb), and Mhem) due to differences in polarizing stimuli, cytokine production, and metabolic peculiarities (Figure 2A) [14,46,47].

Macrophage type prevalence is crucial for atherosclerosis development (Figure 2B). Advanced atherosclerotic plaques were identified in a swine model of atherosclerosis with the injection of M1-polarizing factors [48]. A study on left coronary arteries shows the predominant expression of pro-inflammatory-associated genes in atherosclerotic lesions and the opposite type in the regions devoid of pathology signs [49]. While stable plaques are enriched with anti-inflammatory-type cells in vulnerable plaques, pro-inflammatory macrophages are the most numerous [50,51]. Some data show the preferable accumulation of M2-like macrophages in high-risk unstable plaque. But it may be the result of the demonstrated high heterogeneity of M2-type cells [52]. M1 macrophages are prevalent in rupture-prone regions of plaques [53,54] and are found in the developed plaque during the symptomatic phase; at the same time, M2 macrophages are more present in asymptotic plaques [38]. M2 cells contain fewer lipids compared to M1 and are localized away from the lipid core [14]. Cholesterol crystals may act as M1-polarizing stimuli, exacerbating the cell type ratio in atherosclerotic plaque [55]. Despite the conflicting evidence of the role of metalloproteinases in plaque development, pro-inflammatory and anti-inflammatory macrophages are shown to be greatly different in extracellular matrix-degrading metalloproteinases synthesis [56,57].

The leading role of macrophages in atherosclerosis and other inflammatory diseases as well as the possibility of macrophage phenotype switch [19] explain the perspectives shown in the literature on cell polarization control for atherosclerosis treatment [37,58,59,60,61,62,63,64]. Studies in recent years have identified some molecular targets and endogenous metabolites, which can influence macrophage polarization (carnitine palmitoyl transferases [65], pyruvate dehydrogenase kinase 1 [66], kallistatin [67], parvalbumin [68], N6-methyladenosine (m6 A)-forming enzyme METTL3 [69], angiotensin II, GLUT3 [70], long noncoding RNA PVT1 [71] and Cox2 [72], branched-chain α-ketoacids [73], lactate [74], microRNA miR-657 [75], suppressors of cytokine signaling [76], IFNβ [77], α-aminobutyric acid [78], nucleolin [79], IL-4 [80], IL-17A [81], and IL-6 [82]) as well as a number perspective exogenous biologically active substances (araloside C [83], artesunate [84], berberine [85,86], convallatoxin [87], cynaroside [88], feruloylated arabinoxylan [89], lupeol [82], oleoylethanolamide [90], pinosylvin [91], quercetin [92], resolvin D1 and D2 [93], tannic acid [94], and taurine [95]).

It is widely recognized that the microenvironment creates the conditions of the macrophage polarization route. According to some studies, not only inflammation-associated chemokines but also non-selective metabolic drivers like oxygen and glucose availability, which regulate the balance between basal metabolic pathways, can change the macrophage phenotype profile [64]. But are there any internal cellular triggers defining preferential ways of monocyte differentiation? In our opinion, mitochondrial function is one of the leading internal factors in macrophage polarization. And, therefore, mitochondrial dysfunction due to mtDNA defects can cause disturbances in macrophage polarization and corresponding atherosclerosis resolution. So, the main aim of this review is to find the similarities between the known intracellular consequences of atherosclerosis-associated mtDNA mutations and phenotypic features of pro-atherogenic and anti-atherogenic polarized macrophages.

## 2. Mitochondrial DNA Mutations: Basic Aspects and the Relationship with Atherosclerosis

Mitochondria play a key role in basic processes including synthesis of ATP, calcium homeostasis, fatty acids, amino acids, nucleotides and carbohydrate metabolism, detoxification, and cell proliferation, and result in the regulation of cell death by apoptosis and necrosis [96,97,98,99]. The importance of mitochondria for cell life is based not only on their own function but also because of the close connection with other cellular organelles, primarily with endoplasmic reticulum (ER) [100].

**Figure 3:**
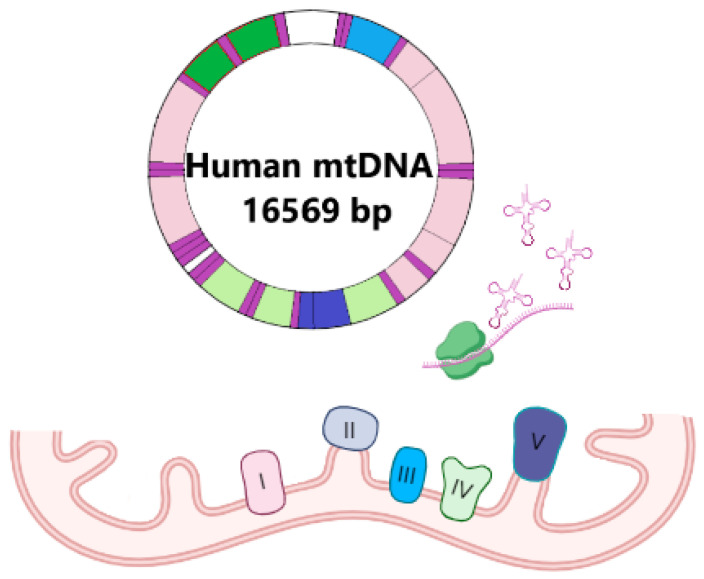
The base of mtDNA mutations influences mitochondrial function. mtDNA encodes subunits of complexes I, III, IV, and V of mitochondrial ETC as well as the machinery of these proteins’ synthesis (12S and 16S rRNAs and all of the tRNAs).

The biology of mitochondria significantly differs from other cell structures due to the presence of its own genome: mtDNA [101]. Human mtDNA (Figure 3) is a double-strand cyclic molecule with a size of 16,569 bp that contains 37 genes [102]. Two of them encode 12S and 16S rRNA, 22—tRNAs, and 13—polypeptides of mitochondrial ETC (seven subunits of NADH dehydrogenase complex (complex I), one subunit of coenzyme Q: cytochrome *c* oxidoreductase (complex III), three subunits of cytochrome *c* oxidase (complex IV), and two subunits of F1-F0 ATP synthase (complex V)) [103,104,105]. Despite the small number of encoding products compared to the whole mitochondrial proteome, which is more than 1500 [106], mtDNA stability is extremely important for mitochondrial function.

Human cells have tens to hundreds and even thousands of mtDNA copies; the presence of identical mtDNA copies in a nucleotide sequence is called homoplasmy. But close proximity to ROS production sites of ETC, a relatively low effective reparation system, absence of histones or similar proteins, and intensive replication cause a high frequency of persistent mutations [107,108,109,110]. As a result, cells and even individual mitochondria can simultaneously contain wild-type and mutant mtDNA, which is called heteroplasmy [111,112]. Because of the high density of exon accumulation, mtDNA mutations can be critical for mitochondrial metabolism and cell life. The higher the heteroplasmy level, the more significant the influence of mutation on mitochondrial function [113]. mtDNA mutations are associated with a significant number of mitochondrial diseases, which are characterized by mitochondrial function disturbances and primarily affect organs and cells with high energy consumption. As a rule, pathologies associated with mtDNA mutations can be observed after the achievement of 60–90% heteroplasmy [114,115,116], but the threshold level depends on the exact mutations and their combinations.

Mitochondrial disturbances including those associated with mtDNA mutations are linked with inflammatory pathologies, among them cardiovascular diseases [24,117,118,119,120,121,122,123,124]. mtDNA mutations have been found in circulating blood cells and atherosclerotic plaques as well [125,126,127,128,129], indicating that mitochondrial genome lesions are part of the atherosclerosis process. Compared to healthy donors, patients with coronary artery disease have significantly increased heteroplasmic variant numbers in the control region of mtDNA, mtDNA deletions, and single nucleotide variants [130]. The m.4435A>G mutation in mitochondrial tRNA^Met^ alters RNA structure and function and decreases efficiency in aminoacylation, leading to arterial hypertension [131]. tRNA^Thr^ m.15927G>A mutation abolishes the highly conserved base pairing (28C-42G) of the anticodon stem, with a subsequent decrease in respiratory rate, mitochondrial membrane potential, and increase in ROS production, which has been shown to lead to coronary artery disease [132]. Estimation of blood cells of patients allowed the identification of four mtDNA mutations (m.3256C>T in *MT-TL1* gene, m.12315G>A—*MT-TL2*, m.13513G>A—*MT-ND5*, and m.15059G>A—*MT-CYB*) associated with high risk of atherosclerosis [133]. By using high-resolution B-mode ultrasonography, it has been shown that these mutations are correlated with typical atherosclerosis carotid intima-media thickness changes [134,135]. Heteroplasmy of m.5178C>A and m.1555A>G mutations has been positively correlated with left ventricular hypertrophy [136]. Age-related accumulation of mtDNA nucleotide deletions linked with respiratory stress has been shown in coronary atherosclerotic disease patients [126]. One of the deletions—mtDNA^4977^—has been closely linked to the death of patients with coronary artery disease [137]. An increased rate of mtDNA heteroplasmy for coronary artery disease was found in the case of three RNA mutations (m.5592A>G, m.5628T>C, and m.681T>C) [138]. Extensive population studies using different probes (blood cells, aorta samples, and atherosclerotic plaques) allowed researchers to find mtDNA mutations with the opposite influence on atherosclerosis progression: pro-atherogenic and anti-atherogenic [139,140,141].

Despite the prevailing opinion about mtDNA mutations as a trigger but not a consequence of atherosclerosis pathology, the exact mechanism of this relationship remains unknown. Most studies have linked the disease progression with the structure and functional defects or the rate of ETC subunit synthesis [127,142], which correlate with decreased OXPHOS efficiency and a significant increase in ROS production, leading to oxidative stress. It is interesting to note that the authors explain the possible anti-atherogenic effect of some mtDNA mutations with the higher stability of the encoded macromolecules compared to wild-type proteins [140,142], but this needs to be verified experimentally. The results of some studies have shown a complex mechanism of pathology, depending on the exact mutation load. For example, in an ApoE^-/-^/polG^-/-^ murine model, atherosclerosis progression was associated with defects of ETC function without an increase in electron leakage from ETC [143]. A possible explanation of the link between atherosclerosis and mtDNA mutations lies in the field of inflammation through the activation of Toll-like receptors (TLRs) [144] or NLRP3 inflammasomes [55] by circulating mtDNA or escape from damaged mitochondria as a result of autophagy or mitochondrial permeability transition pore (mPTP) opening [145,146]. NLRP3 inflammasome activation also can be linked with the change in the function of NAD-dependent deacetylase sirtuins [147] due to the inhibition of mitochondrial respiration and defects of ETC complex I leading to NAD/NADH ratio decrease [148].

According to the inflammation-based relationship between mtDNA mutations and atherosclerosis, the immune system cells, primarily monocytes and macrophages, are the focus of attention. Based on the information above, the question of “whether the atherosclerosis stimulating role of these mutations is linked with the influence on macrophage polarization?” seems to be highly important. The answer to this question can be found by examining the relationship between macrophage biology and changes in mitochondrial metabolism that are associated with mtDNA mutations.

## 3. Mitochondrial DNA Mutations as a Possible Regulator of Cellular Metabolism Associated with Different Types of Macrophage Polarization

**Figure 4:**
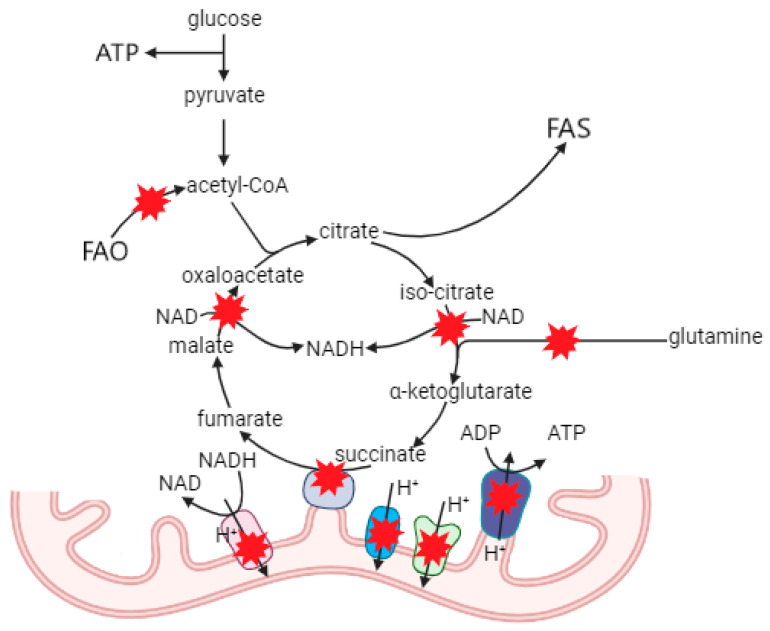
Possible mechanisms of mtDNA mutations that influence basal metabolic processes. Dysfunction of complexes I, III, IV, and V is associated with defective proteins. Complex II, through global alterations in ETC, prevents some reactions of the TCA cycle due to depletion of NAD or accumulation of succinate, which decreases mitochondrial respiration, ATP synthesis, glutaminolysis, and FAO rate. Increased citrate is used for FAS. Energy depletion enhances the role of glycolysis in ATP production.

Macrophage types vary greatly in the function of basal metabolic processes (Figure 2A and Figure 4) as shown in a number of reviews [58,149,150,151,152,153,154].

The most well-known features of M1-type cells include at least two broken points in the tricarboxylic acid (TCA) cycle, increased fatty acid synthesis and decreased fatty acid oxidation, reduced dependence on glutaminolysis, and the higher role of glycolysis in ATP production compared to OXPHOS. As a rule, M2 macrophages are characterized by an inverse metabolic profile. It is obvious that mitochondrial function regulates all of the processes mentioned [155,156,157] that make mtDNA mutations possibly responsible for metabolism changes and the macrophage polarization route. For example, many of the pathological mtDNA mutations including those associated with atherosclerosis lead to a significant decrease in ATP level [127,136,142,158,159,160,161,162,163,164], which relates to the shift from OXPHOS to glycolysis in the case of the mutations m.3243A>G (*MT-TL1*) [165], m.3842G>A (*MT-ND1*) [166], m.1782G>A (*MT-RNR2*), and m.8133C>T (*MT-CO2*) [167]. Less is known about the possible link between macrophage types and changes in mtDNA associated with disturbances in mitochondrial dynamics, intracellular calcium homeostasis, redox balance, and mitochondrial autophagy processes.

### 3.1. mtDNA Mutations and Disturbances in Mitochondrial Dynamics

Mitochondrial activity is under the control of mitochondrial dynamics processes, including fission, fusion, and mitophagy. The main fusion-regulating proteins include mitofusins 1 and 2 (Mfn1 and Mfn2) and Opa1, while mitochondrial fission is regulated by Drp1, Fis1, Mff, MiD49, and MiD51 [168]. Disturbances in the balance between these processes lead to changes in mitochondrial network morphology, for example, fragmentation, elongation of mitochondria, or enhancement of branch point formation.

According to the literature, mitochondrial dynamics plays a significant role in macrophage metabolism, including activation and polarization processes (Figure 1). It has been shown that while M1 macrophages contain mostly fragmented mitochondria, the mitochondrial network of M2-type cells is more developed. An increase in mitochondrial fusion during the transformation of M1 macrophages into the M2 type can reduce inflammation levels in several diseases, including atherosclerosis [169,170].

An important role of mitochondrial fission regulating protein Drp1 in pro-inflammatory macrophage stimulation has been shown in studies with LPS [171]. More than that, Drp1 leads to a pro-inflammatory macrophage phenotype even in the absence of lipopolysaccharide (LPS), while Drp1 silence significantly decreases M1 polarization [172]. At the same time, Drp1 enhances efferocytosis [173], activates macrophages, increases the rate of intima thickening, and, as a result, promotes acute inflammation after the mechanical damage of blood vessel walls [174]. According to the study of Bang et al., stimulation by LPS leads to the interaction of outer mitochondrial membrane phosphatase PGAM5 and Drp1, shifting macrophage polarization to a pro-inflammatory phenotype. Experimental data show that PGAM5-Drp1 signalization is associated with metabolic reprogramming, including regulation of glycolysis and mitochondrial metabolism [175]. In a study on the inhibition of Drp1 by Mdivi-1, the increase in atherosclerosis development was associated with M1-type macrophage polarization [176].

Among the proteins of mitochondrial fusion, the role of Mfn2 is mostly studied. While expression of Mfn2 has increased in wild-type macrophages as well as brain and cardiac cells [177], in ApoE^-/-^ mice, the expression of Mfn2 was significantly reduced during atherosclerosis progression [178]. Also, Mfn2 (but not Mfn1) positively regulates the M1 polarization route by activation of HIF1α [179]. Using the murine model of Mfn2 KO, this protein was shown as necessary for enhancing the pro-inflammatory response of macrophages as the result of aerobic glycolysis induction by HIF1a, which was activated by complex I of ETC and an elevated level of ROS production [177]. Moreover, in a mouse kidney study, higher macrophage polarization into the M2 phenotype was detected as a result of the Mfn2 deficit [180]. The role of Mfn2 in atherosclerosis progression can also be associated with the increase in expression of the cellular carrier of cholesterol in macrophages by activation of PPAR-γ and inhibition of several elements of the MAPK/ERK routes [181]. It should be noted that genetic deletion of Mfn2 enhances pro-inflammatory response by activation of TLR2, as well as NF-κB signaling, which is associated with increased expression of a complex IRAK4 [182]. These study results indicate that the significantly complex character of the macrophage polarization process is dependent on a number of factors.

Another regulating mitochondrial fusion process protein—Opa1—can also influence the metabolism changes in macrophages. Opa1 deletion in macrophages leads to disturbances in signaling routes, metabolic adaptation, and M1-type polarization, as well as the increase in the intracellular concentration of TCA metabolites and defective activation of the NF-κB route [183]. The decrease in Opa1 expression in mice with MIC26 KO is closely related to atherosclerosis progression, including necrotic core growth due to efferocytosis stimulation [184].

According to a number of studies, mtDNA mutations, including those associated with atherosclerosis, can affect the processes of mitochondrial dynamics processes [185]. There is evidence that mtDNA replication is associated with mitochondrial fusion. This process is necessary to maintain the integrity of mtDNA by stabilizing the number of copies, reducing point mutations and deletions, and attenuating the harmful effects of existing mutations [186]. In mice with Drp1 deletion resulting in defective mitochondrial fission, mtDNA tends to aggregate in abnormal structures [187]. In mice with deletion of Mfn1 and Mfn2, a decrease in mtDNA in muscle cells as well as a fast increase in point mutations and deletions in mtDNA were associated with mitochondrial dysfunction and high lethality [186]. The shift of the balance from the M2 to M1 macrophage type with mitochondrial dysfunction development and a higher level of response to infections has been shown in mice with PolG mutations (PolGD257A), which leads to the accumulation of mtDNA mutations [188]. It should be noted that the release of mtDNA into the cytosol is another negative factor for mitochondrial dynamics. Defects of mtDNA with release into the cytosol, leading to an inflammatory response in a number of metabolic diseases with chronic inflammation, have been shown to be a result of mitochondrial dysfunction due to overexpression of Drp1 [185,189].

### 3.2. mtDNA Mutations and the Changes in Selective Mitochondrial Autophagy

Mitophagy is one of the key processes in the maintenance of mitostasis, which removes defective mitochondria. To date, two main mitophagy mechanisms—PINK1/Parkin- and receptor-mediated mechanisms—are the most studied [24,190]. They are closely related to mitochondrial dynamics and require an increase in mitochondrial fission for the subsequent lysosome-based degradation of fragmented organelles [191]. Defected mitophagy has been shown as an element of atherosclerosis pathogenesis and inflammaging [192,193], which indicates the importance of understanding the possible influence of mitophagy on macrophage polarization and the dependence of the process on mtDNA disturbances (Figure 2).

M1 macrophages derived from the THP-1 cell line exposed to low-dose LPS/IFNγ exhibit a lower colocalization of lysosomes and mitochondria, relative to M0, indicating reduced mitophagy activity [95]. In vivo experiments show that macrophages exhibited the M1 phenotype and displayed a lower level of mitophagy in the kidneys of streptozocin-induced diabetic rats [194]. Patoli et al. show that LPS/INFγ inhibited PINK1-dependent mitophagy, which triggered classical macrophage activation (M1) as well as mitochondrial uncoupling, and mitophagy stimulation led to the reversal of the classical polarization route [195]. Enhanced M1 and impaired M2 polarization associated with reduced activity of AMPK and mitochondrial biogenesis has been shown for macrophages from rats with chronic kidney disease [196].

THP-1-derived macrophages treated with Th2-like cytokine IL-25 induce mitophagy through the ROS-AMPK pathway to stimulate M2 macrophage polarization [197]. In macrophages derived from the THP-1 cell line, it has been shown that arsenic leads to the polarization and repolarization of macrophages into the M2 type through activation of mitophagy by microtubule-associated protein 1 light-chain 3 and phosphor-Parkin protein markers [198]. In a THP-1 cell study, M2 macrophage polarization was associated with acrylamide-based increase in ROS generations and PINK1 expression [199]. Increased production of M2- and a decrease in M1-related cytokines were shown in THP-1 cells with upregulation of PINK1/Parkin and LC3 through the AMPK pathway due to the influence of IL-33 [200]. The shift in balance from M1 to M2 macrophage-mediated mitophagy promotion is one of the explanations for the anti-atherosclerosis and anti-hepatic fibrosis capabilities of the herbal medicine Ger-Gen-Chyn-Lian-Tang [201]. Macrophages derived from LPS-induced RAW264.7 cells treated with paeoniflorin showed suppressed renal inflammation by promoting macrophage polarization from M1 to M2 and inducing mitophagy via regulating KLF4 [202]. In a LUAD cell study, M2 macrophage polarization positively correlated with increased mitophagy due to PFN1 depletion [203]. Regulation of mitochondrial metabolic enzyme MTHFD1L leading to increased expression of autophagy-related proteins was associated with suppression of macrophage polarization in M1 [204].

So, all of the studies cited link decreased mitophagy with M1-type macrophage polarization. Despite the prevalence of information about M2 macrophages' association with a higher level of defective mitochondria removal, some controversial data are presented. BM-derived macrophages that lost PINK1 or Parkin resulted in an increased frequency of profibrotic/M2 macrophages [205]. In a bleomycin-induced pulmonary fibrosis oxidative stress study, mitochondrial dysfunction and M2 macrophage polarization were associated with PINK1/Parkin downregulation, while the promotion of mitophagy by PINK1 overexpression reversed the macrophage phenotype [206]. Unfortunately, in these studies, there are no data about alternative mitophagy pathways in macrophages involved in fibrosis, which leaves the question about the possibility of decreased mitophagy in M2 macrophages open.

A literature analysis showed a close relationship between mtDNA mutations and changes in mitophagy levels. mtDNA-mutator mice with the defective proofreading function of polG were unexpectedly characterized by insufficient autophagy-based clearance of damaged mitochondria [207]. In m.3243A>G mutated cells and MELAS patient fibroblasts, the mutation was associated with a reduced expression of ATAD3B, a novel mitophagy receptor, and a subsequent decrease in damaged mitochondria clearance level [208]. Rhabdomyosarcoma cells with the same mutation showed a negative correlation between mutant load and markers of mitophagy expression [209]. A study of MELAS patients' cells with mtDNA mutations showed increased autophagy but not mitophagy processes, indicating insufficiency of mtDNA existence for dysfunctional mitochondria clearance [210]. Surprisingly unelevated autophagy levels were detected in osteosarcoma nuclear genetic background cell lines with partial deletion of mtDNA and mutations m.3243A>G and m.8993T>G compared to cells with wild-type mtDNA [211]. Defected basal and stimulated PINK1/Parkin mitophagy has been shown for monocytes containing mutations associated with atherosclerosis [212,213,214]. Zhang et al. detected PINK1/Parkin-dependent mitophagy in cybrids with the m.3460G>A mutation [215].

### 3.3. mtDNA Mutations and Changes in Intracellular Calcium Homeostasis

Ca^2+^ homeostasis is under complicated control of several membrane ion channels and Ca^2+^-ATP-ases [216,217] and intracellular calcium stores, including ER and mitochondria [218]. Disturbances of this system are responsible for atherosclerosis progression [219,220,221,222]. One of the possible mechanisms linked with mitochondrial dysfunction in a calcium-dependent manner is due to changes in the intracellular calcium concentration ([Ca^2+^]_i_) and the activity of some mitochondrial enzymes or an increase in mitochondrial permeability and cell apoptosis [124,223]. For example, ER stress-associated mitochondrial alterations were shown in a PON2-def/ApoE^−/−^ murine model of atherosclerosis [224]. Moreover, disturbance of the intracellular Ca^2+^ homeostasis system of key players of the atherosclerotic process (endothelial cells, VSMCs, and macrophages) is responsible for the induction of calcium deposition in the vascular wall, decreasing elasticity of aorta and large arteries, and pathological hemodynamic alterations [225]. It has been shown that microcalcifications lesser than 50 µm in size accumulating in unstable plaques are specific for pro-inflammatory pro-atherogenic macrophages [226,227]. But is there a relationship between macrophage polarization and alterations in Ca^2+^ homeostasis associated with mtDNA mutations?

Macrophage types differ in the basal Ca^2+^ level in the cytosol as well as in organelles. Thus, pro-inflammatory stimulation by LPS, leading to the formation of M1 macrophages, induces [Ca^2+^]_i_ increase in cells [228]. On the other hand, the change in [Ca^2+^]_i_ demonstrates the polarization effect itself. While the increase in [Ca^2+^]_i_ leads to the polarization of macrophages into M1-type cells, [Ca^2+^]_i_ decrease is associated with the inverse change in which the M2 phenotype is positively correlated with the regeneration of the bone tissue damage [229]. It is important to note that the polarizing effect is not only determined by the Ca^2+^ concentration change but also by the type of calcium-permeable channel or transport system of the plasmalemma as well as the ER and mitochondrial membranes [230,231].

Despite the fact that the level of Ca^2+^ in ER, as well as the influx of Ca^2+^ into cells, is higher in polarized macrophages compared to naïve cells, the exact macrophage phenotype is associated with the function of different Ca^2+^-permeable channels: TRPC1 for M1 and Orai1 for M2 [228]. Macrophage repolarization is associated with the change in the expression level of channels; LPS stimulation shifts M2 cells from Orai1 toward TRPC1-mediated Ca^2+^ entry. Moreover, blockage of Ca^2+^ entry leads to a decrease in the polarization in both pathways. Another experimental study also showed that the M1 polarization pathway is mostly dependent on TRPC1 function. Infection of TRPC^-/-^ mice led to inhibition of inflammatory mediator expression [232].

Ca^2+^-sensitive receptor (CaSR) demonstrates the ambiguous role in the macrophage polarization process. This receptor, coupled with G-protein, in particular G_i/o_ and G_q/11_ [233], analyzes extracellular Ca^2+^ concentration. Pharmacological activation of CaSR increased [Ca^2+^]_i_ in RAW264.7 macrophages, suppressing macrophage polarization into type M1 [234]. Another macrophage study showed Ca^2+^-dependent enhancement of the transcription of M2-type macrophage markers (Arg1 and IL-10) as a result of CaSR activation [235]. The opposite effect has been shown using mouse peritoneal macrophages: activation of CaSR led to an increase in [Ca^2+^]_i_ due to the function of TRPV4 channels. The effect was associated with activation of PLA2/CYP450 and PLC/PKC signaling cascades and resulted in preferential M1 macrophage polarization [236]. The same results were shown on THP-1 cells: CaSR stimulation was associated with enhanced expression of pro-inflammatory markers and led to activation of inflammasome NLRP3 [237].

Human macrophages are characterized by the expression of different types of metabotropic and ionotropic purine receptors, including P2Y_1_, P2Y_2_, P2Y_11_, P2X_1_, P2X_4_, and P2X_7_. Activation of these receptors (primarily P2Y_2_) by extracellular ATP and UTP led to [Ca^2+^]_i_ oscillations and expression of IL-6, indicating pro-inflammatory polarization of macrophages [238]. Unfortunately, this effect does not depend on the activity of PKC, NFAT, and MAP-kinase p38. Moreover, M1 and M2 macrophages differ in the expression level of purine receptors. P2X receptors (mostly P2X7) are highly exhibited on the membranes of M2 macrophages, and the deficiency of these receptors impairs the M2 polarization route [239]. In the study of Zumerle et al., intercellular communication via ATP was associated with P2X4 and P3X7 receptors, which are more specific for pro-inflammatory macrophages [240].

Experiments with primary cultures of human monocyte-derived macrophages showed a significant role of MCU for macrophage polarization. MCU inhibition or knockdown was associated with reduced calcium uptake, M2 polarization, and macrophage phagocytic ability and, at the same time, with no effect on the M1 polarization route [241].

**Figure 5:**
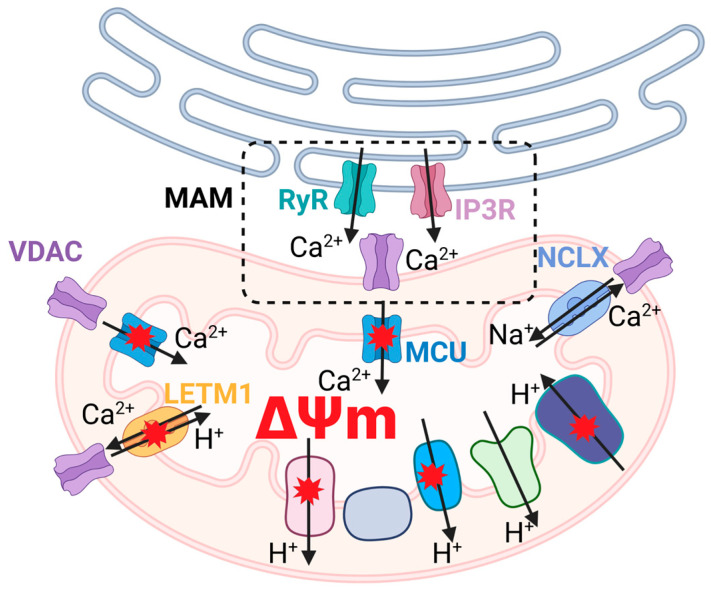
mtDNA-associated mitochondrial dysfunction and disturbances of intracellular calcium homeostasis. Mitochondria calcium buffering is primarily supported by MCU transportation of Ca^2+^ into the matrix due to the electrochemical gradient that is mostly formed by the mitochondrial membrane potential (ΔΨm). Efflux of Ca^2+^ from the matrix is provided by the mitochondrial Na^+^-Ca^2+^ exchanger (NCLX) and leucine zipper–EF hand-containing transmembrane protein 1 (LETM1). ETC dysfunction is frequently linked with ΔΨm decrease, which leads to alterations in calcium buffering capability. Complex V dysfunction reduces the energy support role of calcium transport processes. The close relationship of mitochondria with ER (Ryanodine (RyR) and inositol trisphosphate (IP3R) receptors) worsens ER stress due to a decrease in MAM formation.

Maintenance of Ca^2+^ homeostasis is a highly energy-consuming process. Because ATP production is one of the most important functions of mitochondria, mtDNA-associated disturbances in mitochondrial metabolism can be critical for Ca^2+^-dependent processes (Figure 5), including macrophage polarization. Age-related mtDNA mutations can be the cause of disturbances in mitochondrial Ca^2+^ uptake in macrophages due to a decrease in the expression of MCU and its regulatory subunit MICU1 [242]. The m.4263A>G mutation in the tRNA^Ile^ gene associated with maternally inherited hypertension was linked with Ca^2+^ homeostasis disturbances, including decreased mitochondrial Ca^2+^ as well as a lower level of MCU expression [243]. A number of mtDNA mutations associated with MELAS, LHON, and MERRF were shown to be responsible for the high cell sensitivity to oxidative stress due to calcium deregulation and mPTP opening [244]. A significant decrease in mitochondrial Ca^2+^ uptake was detected in cybrids with the m.13565C>T mutation in MT-ND5, leading to impaired bioenergetic function [245]. The m.3243A>G mutation, which led to the change in glucose metabolism, impairment of OXPHOS, and ROS production, was associated with an increase in stimulated calcium amplitude [161]. Elevated cytosolic calcium has been shown to be a result of the influence of the pathological m.2336T>C mutation in the mitochondrial 16S rRNA gene [149]. Another possible mechanism of the relationship between mtDNA mutations and calcium homeostasis disturbances is linked with increased ROS production in the mitochondrial matrix and cytosol [246,247,248], which is discussed further in this article.

### 3.4. mtDNA Mutations and the Redox Balance Shift

A body of evidence suggests that oxidative stress plays an important role in the initiation and progression of atherosclerosis [249]. Recent investigations into the pathogenesis of the atherosclerotic process have focused on the "oxidative modification hypothesis" of the pathology [250,251]. According to this hypothesis, the oxidation of LDLs plays a central, if not obligatory, role in the pathogenesis of atherosclerosis. Currently, a significant amount of evidence supports the hypothesis that oxLDLs play a role in the pathophysiology of both the initiation and progression of atherosclerotic lesions through various mechanisms, including their pro-inflammatory, immunogenic, and cytotoxic properties [252].

Redox balance between the production and scavenging of ROS plays a critical but contradictory role in the macrophage polarization process and metabolism [253]. In particular, ROS play a role in enabling macrophage survival under hypoxic conditions, stimulating metabolic shift to a more glycolytic phenotype [254,255].

In the context of the role of redox balance in macrophage physiology, it is useful to analyze different sites of ROS production and differentiate the influence of ROS on the macrophage polarization route and ROS-producing activity of already polarized cells.

The intracellular redox balance is under the control of a complicated system of ROS-producing sources and antioxidant systems, including enzymes and low-molecular-weight substances [256,257,258].

As a rule, M1 macrophages are considered as more ROS-producing (primarily in the form of O2−·) cells by ETC as well as NOX family enzymes compared to M2 [253,259,260,261]. At the same time, M2 macrophages are characterized by a lower rate of ROS production due to reduced levels of NOX enzymes as well as increased expression of antioxidant enzymes, including Cu/Zn-SOD, Gpx1, and catalase [262]. But it should be noted that due to the abovementioned high diversity of polarized macrophages, there are some exceptions. For example, increased O2−· production has been shown for IL-4-stimulated cells [263].

A number of studies consider H_2_O_2_ and H_2_O_2_-producing enzymes as important triggers of the M2 macrophage polarization route [262,264,265,266,267]. Increased production of H_2_O_2_ by Cu,Zn-SOD modulates STAT6 transcription factor activity and regulates Jumonji domain-containing 3 with subsequent forming of M2-type macrophages [264,265]. The mechanism of M2 polarization includes STAT6 activation via the oxidation of cysteine residues by SOD1-dependent H_2_O_2_ [264]. In a lung fibrosis study, increased Cu,Zn-SOD expression was associated with activation of profibrotic signaling pathways and enhanced pathology processes involving M2 phenotype macrophages [267]. These data correlate with the known role of Mn-SOD activity in the atherosclerosis process. Depletion or reduction in Mn-SOD is associated with an M1 macrophage phenotype increase of the necrotic core and vulnerability of atherosclerotic plaque, while elevating Mn-SOD activity leads to an anti-atherosclerosis effect [266].

The defining role of mitochondrial ROS increase in promoting macrophage reprogramming towards the M1 phenotype has been shown by different studies [253,259,260], which can explain the anti-atherosclerosis effect of mitochondrial O2−· scavengers [266]. At the same time, augmented mitochondrial ROS production and induced mitochondrial biogenesis positively correlate with lung macrophage profibrotic (M2) polarization [268]. Also, increased mitochondrial ROS production after ATP synthase partial inhibition triggers an anti-inflammatory response with M2 macrophage polarization, which can be prevented by the mitochondria-targeted antioxidant MitoQ [269]. The difference shown indicates the opposite influence of mitochondrial ROS types, while increased O2−· stimulates the M1 phenotype and a higher level of H_2_O_2_ is important for M2 macrophage polarization.

The NOX family, predominantly NOX2, is considered one of the main ROS production sites in polarized macrophages [261]. Studies show increased expression of NOX proteins as well as activity of these enzymes [261]. NOX inhibitors decrease pro-inflammatory cytokine release by LPS-induced macrophages [270]. Also, decreased ROS production during the transition from the M1 phenotype to the M2 phenotype was associated with the inhibition of NOX2 [271]. Downregulation of macrophage response to LPS and IL-4 due to NOX2 inactivation has been shown in different studies [271]. In injured cortex studies of NOX2^-/-^ mice, a reduction in STAT1 and an increase in STAT3, but not STAT6 signaling, and a decrease in pro-inflammatory markers release led to less polarization in the M1 macrophage phenotype [272]. This finding can explain the results of increased fibrous cap thickness, less necrotic core formation, and enhanced efferocytosis by regulating the MertK/PI3K/AKT pathway as a result of NOX2 inhibition in a murine-vulnerable carotid plaque model [273].

The data above show a negative correlation between NOX2 activity and the M1 polarization route. At the same time, some studies demonstrate the opposite results. In a murine model of Japanese encephalitis, NOX2 silencing as well as oral administration of ROS scavengers was associated with a more pronounced formation and accumulation of M1 macrophages [274]. Pharmacological NOX2 inhibition decreased M2 macrophage polarization from human blood monocytes [275]. In a tumor-associated macrophage study, NOX1 and NOX2 activity was shown as critical for the M2 polarization process. Silencing of the enzymes led to impairment of macrophage differentiation [276]. While comparing the data with NOX2 inhibition, it is necessary to keep in mind that macrophage types differ in H_2_O_2_ production rate depending on the increase in O2−· level [261].

NOX4 activity has been shown as critical in M2 macrophage polarization. Increased NOX4 activity positively correlates with lung macrophage profibrotic (M2) polarization [268]. NOX4 deficiency has been associated with reduced M(IL-4+IL-13) and forced M(LPS+IFNγ) polarization routes and with higher levels of NOX2 expression [277].

**Figure 6:**
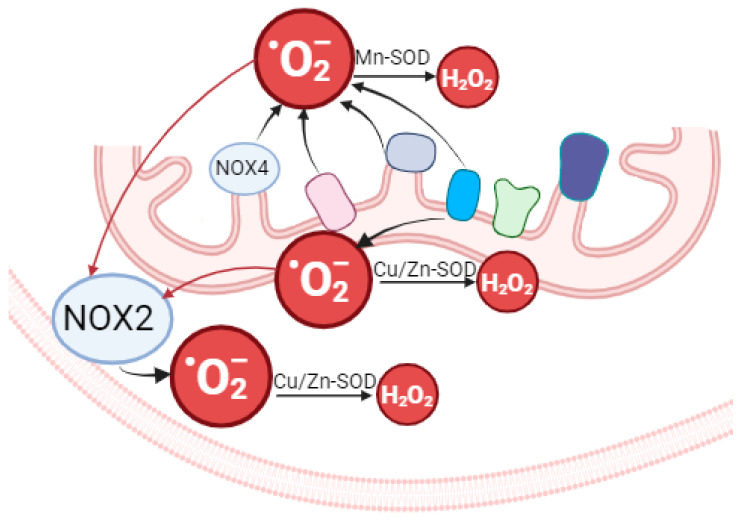
The relationship between mitochondrial and cytosolic ROS in macrophages. ETC dysfunction leads to an increase in the production of O2−· in the mitochondrial matrix (by complexes I, II, and III) as well as in the intermembrane space (by complex III). Transmembrane transporting systems and mPTP opening can provide O2−· release into the cytosol and activation of NOX2.

Recent studies show direct communication between mitochondria and NOX-derived ROS. There is information about the localization of NOXs (mostly NOX4) on mitochondrial membranes [278,279]. But, more importantly, NOXs (primarily NOX2 [246]) and mitochondria are characterized by a cross-talk mediated by ROS [279,280,281,282]. Increasing mitochondria-stimulating NOX2 activity in SOD-deficient models shows the leading role of O2−·, not H_2_O_2_, in the process [246]. Dysfunctional ETC leads to increased production of O2−·, which may be released into the cytosol through inner membrane anion channels or mPTP [283] (Figure 6).

The close relationship between redox balance changes and macrophage polarization presents the possibility of inflammation regulation by inhibition of ROS production. Prevention of monocyte differentiation into M1-type macrophages due to decreased ROS production via the activation of AMPK has been shown to be a result of the anti-inflammatory effect of metformin [284]. In osteoarthritis studies, reduction in intracellular ROS production with restoration of mitochondrial membrane potential and increase in reduced glutathione level were shown as an effective route to repolarize the M1 macrophages to M2 [285].

As shown in a number of studies, most mutations of mtDNA are associated with the change in the balance between production and neutralization of ROS. Thus, atherosclerosis-related mutations (del652G, m.3256C>T, m.12315G>A, m.13513G>A, and m.14459G>A) lead to a deactivation of OXPHOS with an increase in the rate of ROS [286]. Functional experiments showed that the homoplasmic m.4263 A>G mutation in the MT-ND1 gene, which is associated with familial maternally inherited essential hypertension, significantly reduces substrate-dependent oxygen consumption in complex I, III, and IV by 70–80% and increases ROS levels in lymphoblastoid cell lines derived from individuals with this mutation [287]. It has been reported that the m.13513 G>A mutation causes a substitution of the amino acid at position 393 of ND5. This substitution may interfere with the quinone binding site, potentially affecting the function of the OXPHOS system. As a result, mitochondrial dysfunction, an increase in ROS production, and a reduction in ATP synthesis could occur [288,289]. The study in [290] presents the direct link of the m.1555A>G mutation in the 12S rRNA gene to both hearing impairment and hypertension. It has been suggested that to maintain normal respiratory function in lymphoblastoid cells with the m.1555A>G mutation, a reduction of approximately 50% in mitochondrial translation capacity is required. Deviations from this level can cause aberrant mitochondrial respiration, resulting in oxidative stress, decoupling of oxidative pathways for ATP synthesis, and eventual failure in cellular energy processes. The mutation m.15059G>A, which is significantly associated with an increased risk of essential hypertension, leads to reduced production and function of mitochondrial cytochrome b and, as a consequence, affects OXPHOS, decreases mitochondrial oxygen consumption, and increases ROS generation [291]. The mutation of the mitochondrial genome in tRNA^Met^ m.4467C>A results in increased production of ROS in lymphocyte cell lines from patients with hypertension [292]. In cellular models, the effects of mutations affecting the leucine (m.3243A>G) and lysine (m.8344A>G) tRNAs included an increase in ROS production and antioxidant system activity, which subsequently preserved cell viability [293]. A study on hypoxia-associated pulmonary hypertension [294] revealed that the mutation m.3379A>G in the ND1 gene results in elevated ROS production and the destabilization of HIF1α, thereby impeding the ability of the body to adapt to the hypoxic environment. The mutation in the ND5 subunit m.12417insA in cybrid cells C8T and C9T has been observed to increase the rate of O2−· production, with a concomitant increase in SOD1 activity [295].

## 4. Perspectives

This review shows the close similarities between disturbances in mitochondrial function and intracellular processes associated with atherogenic mtDNA mutations, on the one hand, and phenotype features of pro-inflammatory M1-type macrophages, on the other hand. This shows that mitochondria are key internal triggers of atherosclerosis-related inflammation. Confirmation of this hypothesis suggests that targeted pharmacological regulation of mitochondrial function could be a promising tool for controlling macrophage polarization and maintaining a balance between different types of macrophages to maintain homeostasis and prevent pathology progression. But the effectiveness of the exact pharmacological strategy will depend on the combination and heteroplasmy level of mtDNA mutations that require a personalized approach to each patient.

## 5. Conclusions

Despite the well-known fact that the direction of macrophage polarization is associated with external factors, changes in cellular metabolism linked with mitochondrial dysfunction can play the role of an internal trigger. Macrophage phenotypes vary greatly in bioenergetic processes; the rate and source of ROS production; intracellular Ca^2+^ concentration; and the mechanisms of Ca^2+^ homeostasis regulation, mitochondrial dynamics, and mitophagy. The analysis revealed that mtDNA mutations associated with inflammation progression lead to similar changes in studied aspects of mitochondrial function that are common for macrophages of pro-atherogenic type M1. So, pathogenic mtDNA mutations can lead to a definite macrophage polarization direction with a respective influence on atherosclerosis progression.

## Abbreviations

ETC—electron transport chain; LDLs—low-density lipoproteins; oxLDLs—oxidized low-density lipoproteins; MAM—mitochondria-associated membrane; MCU—mitochondrial calcium uniporter; mPTP—mitochondrial permeability transition pore; mtDNA—mitochondrial DNA; NOX—NADPH oxidase; Orai1—calcium release-activated calcium channel protein 1; OXPHOS—oxidative phosphorylation; ROS—reactive oxygen species; TCA—tricarboxylic acid; TRPC1—transient receptor potential canonical channel 1; VDAC—voltage-dependent anion channel; and VSMCs—vascular muscle cells.
